# The use of genotyping in antimalarial clinical trials: a systematic review of published studies from 1995–2005

**DOI:** 10.1186/1475-2875-5-122

**Published:** 2006-12-14

**Authors:** William J Collins, Bryan Greenhouse, Philip J Rosenthal, Grant Dorsey

**Affiliations:** 1 Department of Medicine, University of California, San Francisco, Box 0811, CA 94143, USA

## Abstract

**Background:**

The use of genotyping to distinguish recrudescent from new infections is currently recommended for all clinical antimalarial efficacy trials by the World Health Organization. However, genotyping-adjusted drug efficacy estimates may vary between trials due to the use of different genotyping methods and to the different settings in which these methods are applied.

**Methods:**

A systematic review of all clinical antimalarial efficacy trials published from 1995–2005 was performed to characterize the use of genotyping, including the methods used and the effect of these methods on estimates of drug efficacy.

**Results:**

In a multivariate analysis, the method of interpretation of genotyping results, the studied therapy, the location of the trial, and the duration of study follow-up all had statistically significant effects on the percent of genotyped outcomes classified as new infections.

**Conclusion:**

Criteria for defining appropriate, standardized genotyping methods for use in different settings are needed to enable more accurate estimates of antimalarial drug efficacy and better comparison between trials. The advantages and disadvantages of different genotyping methods and their potential impact in various settings are discussed.

## Background

The spread of antimalarial drug resistance in *Plasmodium falciparum *has led to serious setbacks in global malaria control and stimulated an urgent search for new treatments[1]. Monitoring of antimalarial drug efficacy and policy decision making is generally based on the results of clinical antimalarial trials. To adequately assess response to antimalarial therapy in clinical trials, the World Health Organization (WHO) currently recommends that patients be followed for a minimum of 28 days, as treatment failures may occur a number of weeks after therapy[2]. Antimalarial efficacy trials usually occur in areas where malaria is endemic and, therefore, patients may be treated successfully but newly infected with parasites during the follow-up period. When subjects have recurrent parasitaemia following therapy, it is not possible to clinically distinguish between a recrudescence due to drug failure and a new infection. To make this distinction, molecular genotyping techniques have been used to determine whether recurrent parasitaemia is due to the same parasite strain(s), indicating recrudescence, or different strain(s), indicating new infection.

The use of genotyping in antimalarial trials has become more important in recent years due to longer follow-up periods and the use of more efficacious drugs such as artemisinin combination therapies (ACTs), as both of these factors increase the proportion of subjects with recurrent parasitaemia who have new infections. The WHO now recommends genotyping be used for all antimalarial efficacy trials if available[3]. However, genotyping techniques have not been standardized, results may vary between laboratories[4], and different methods of interpretation may have a large effect on estimates of treatment efficacy[5]. To characterize changing patterns of genotyping use and variations in genotyping methodology, and to assess the effect of various genotyping methods on estimates of treatment efficacy, a systematic review of the use of genotyping in antimalarial clinical trials published from 1995–2005 was performed. The primary goal was to provide a comprehensive framework of prior work to help develop a standardized approach to genotyping.

## Methods

### Search strategy

Two reviewers independently screened citations in the following electronic databases for relevant articles: Medline, Embase, Cochrane CENTRAL Register of Controlled Trials, BIOSIS, Web of Science, and Current Controlled Trials. Search terms included "malaria", "falciparum", "efficacy", "clinical trial", "drug", "therapy", and "treatment". The search was limited to English language papers published between 1995 and 2005. Reference lists from primary and review articles were also searched. Individual authors were contacted for unreported study characteristics and additional data of interest. All published papers were downloaded or copies of the original papers obtained. Unpublished data were not sought.

### Selection criteria

Published studies were included if they fulfilled all of the following selection criteria: 1) treatment efficacy was a reported outcome, 2) primary efficacy results were not reported previously, and 3) human subjects with falciparum malaria were included. Studies restricted to complicated or severe malaria, non-biomedical drugs (i.e. herbal therapy), and prophylactic therapy were excluded. Two independent reviewers judged study eligibility, and disagreements were resolved by consensus.

### Data abstraction

The two reviewers independently abstracted data from included studies using a previously piloted data abstraction form. Disagreements were discussed and resolved by consensus. Reviewers were not blinded to details of the publications. The following information was abstracted from all publications: 1) year of publication, 2) country(ies) where the study was done, 3) maximum duration of patient follow-up, 4) treatment arms, 5) number of patients enrolled in each treatment arm, and 6) whether genotyping was performed to distinguish recrudescence from new infections. For all studies that included genotyping, the following additional information was abstracted: 1) earliest day following therapy on which samples were collected for genotyping, 2) method used to collect blood and extract DNA, 3) genotypic markers used, 4) method of distinguishing PCR products, 5) method used to classify treatment outcomes based on genotyping results, and 6) proportion of patients with various genotyping adjusted outcomes for each treatment arm. For two studies, genotyping results were not published in the primary paper but were reported in subsequent publications. These studies were included as having performed genotyping.

### Data management and statistical analysis

All papers identified were entered into an EndNote Library (Thomson ResearchSoft). Abstracted data were double-entered into Microsoft Access and analysed using Stata version 8 (Stata Corporation, College Station, TX.). Count data were analysed using a Poisson regression model. Categorical variables were compared using the Chi-squared test. Univariate and multivariate analyses of the association between various explanatory variables and the proportion of genotyped recurrences classified as new infections were done using logistic regression.

## Results

### All antimalarial trials

A total of 384 studies published between 1995 and 2005 were identified that included estimates of efficacy for the treatment of uncomplicated falciparum malaria. Temporal trends in the number of published studies varied according to the region of the world where they were performed (Figure). In Africa, there was no significant change in the number of studies published per year from 1995–2000 (mean 15; p = 0.71), but the number increased steadily from 15 in 2001 to 43 in 2005 (p < 0.0001). In contrast, there has been no significant change in the number of studies published annually from South and East Asia (mean 11, p = 0.89) or the rest of the world combined (mean 4.5, p = 0.16).

### Antimalarial trials using genotyping

**Figure 1:**
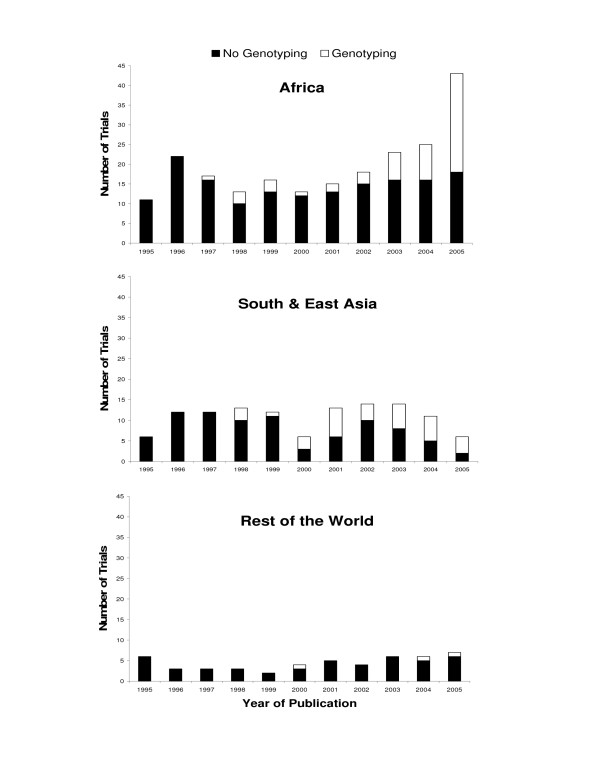
**Annual number of trials with and without genotyping by region**. Total bar height represents all trials. White represents trials that used genotyping. Black represents trials that either did not use genotyping or did not have any episodes of recurrent parasitaemia to genotype.

The main interest of this study was to investigate the use of genotyping in antimalarial clinical trials. Considering all studies included in this review from 1995–2005, 91 (24%) used genotyping to adjust estimates of treatment efficacy, 269 (70%) did not use genotyping, and 24 (6%) had no episodes of recurrent parasitaemia. Temporal and geographic trends in the proportion of studies that used genotyping were similar to trends in the number of studies as a whole (Figure 1). The first study to report the use of genotyping to adjust estimates of treatment efficacy was in 1997 from Africa. Between 1997 and 2002, the overall proportion of studies from Africa that used genotyping was 14% and did not change significantly over this time period (p = 0.76). From 2003–2004 the proportion of studies from Africa that used genotyping increased significantly to 33% (p = 0.008) and further increased to 58% (p < 0.0001) in 2005. For studies conducted in South and East Asia, genotyping was first reported in 1998 and was used in 16% of studies published between 1998 and 1999. Since 2000, approximately 50% of studies from south and east Asia have used genotyping, with no significant change in this proportion over time (p = 0.63). Considering studies done outside of Africa and south-east Asia, none of 17 studies published prior to 2000 and only three of 32 studies (9%) published since 2000 reported using genotyping. The use of genotyping was also associated with studies that included ACTs and studies with longer durations of follow-up. Considering studies published after 1999, 45% of studies that included ACT regimens used genotyping compared to less that 4% of studies that did not include ACT (p < 0.0001). Similarly, 57% of studies that followed patients 28 days or longer used genotyping, compared to 23% of studies that followed patients for less than 28 days (p < 0.0001).

### Genotyping methods

The 91 trials which used genotyping were investigated to determine methods and assess the effects of variations in methods on the classification of outcomes. Genotyping relies on the genetic diversity present in *P. falciparum *to distinguish whether recurrent parasitaemia after therapy is due to recrudescence of the initial parasite strain or to infection with a new strain. To make this distinction, blood samples are collected at baseline and then at the time of recurrent parasitaemia, and parasite genotypes from these two time points are compared. Because *P. falciparum *is haploid in the human host and genotyping markers are single-copy genes, each different allele detected by a genotyping marker represents a genetically distinct parasite strain. If the baseline and recurrent parasitaemia samples have matching alleles, recurrent parasitaemia is classified as recrudescence; if the two samples have different alleles, recurrent parasitaemia is classified as a new infection. The genotyping methods discussed below have varied ability to measure the genetic diversity of parasites. Greater ability to measure diversity increases the sensitivity for detecting new infections, as it is less likely that two different parasite strains will have a matching allele by chance. Methods at all stages in the genotyping process, from when samples were collected to the interpretation of results, varied widely between the 91 trials that were assessed.

### Sample collection and DNA extraction

Collection of baseline and recurrent parasitaemia samples was evaluated first. All 91 trials collected blood samples prior to antimalarial therapy to determine the *P. falciparum *alleles present at baseline, and two trials collected additional samples on subsequent days (one or three days after the onset of therapy) to broaden their definition of the baseline alleles. Most trials only genotyped samples from episodes of recurrent parasitaemia that occurred after a defined number of days following therapy. Episodes before this defined cut-off were assumed to be due to recrudescence. There was large variation in this cut-off between trials, with trials evenly divided into three groups – those with a cut-off less than one week (34%), at one week (36%), or greater than one week (30%). The advantage of a later cut-off is that fewer samples need to be genotyped. However, if new infections occur before this cut-off they are incorrectly classified as recrudescence, leading to a falsely elevated drug failure rate. Of the nine trials which genotyped within one week following the onset of therapy and reported the day of their first new infection, three (33%) had at least one new infection within the week after therapy.

There was less variation in the type of sample collected (Table 1). Most trials used spots of dried blood on filter paper as the source of DNA, a method of collection which requires a small amount of blood, obtainable from a finger prick, and which results in samples which are easy to store and transport. DNA from samples was extracted primarily using common laboratory reagents. Chelex extraction[6] was the most commonly reported DNA extraction method, followed by standard phenol-chloroform extraction, with only 12% of studies reporting the use of more expensive commercial purification kits. Chelex extraction has the advantage of being inexpensive and quick and easy to perform, and it does not require toxic reagents.

**Table 1:** Characteristics of drug trials where genotyping was performed (n = 91)

**Characteristic**	**N (%)**
Source of sample	
filter paper	70 (77%)
whole blood/RBC pellet *	12 (13%)
not reported	9 (10%)
Method of DNA extraction	
Chelex	38 (42%)
phenol/chloroform	17 (19%)
commercial kit	11 (12%)
methanol	1 (1%)
not reported	24 (26%)
Markers used	
*msp1 *alone	2 (2%)
*msp2 *alone	20 (22%)
*msp1 *and *msp2*	19 (21%)
*msp1*, *msp2 *and *glurp*	46 (51%)
*msp1*, *msp2 *and others ^†^	2 (2%)
not reported	2 (2%)
Interpretation of mixed results ^‡^	
always a recrudescence	56 (62%)
always a new infection	17 (19%)
> half of RP alleles match baseline ^§^	5 (5%)
not reported	13 (14%)

* One study used either filter paper or whole blood.

^† ^One study used *msp1*, *msp2 *and *csp*. Another used *msp1*, *msp2*, *glurp*, *trap*, and *pf60.1*.

^‡ ^A mixed result is defined here as a recurrent parasitaemia sample which shows both alleles that were present in the baseline sample as well as new alleles not seen at baseline.

^§ ^RP = Recurrent parasitaemia sample. Four of five studies used *msp2 *alone and defined recrudescence as > half of RP alleles matching alleles found in the baseline sample. One study used *msp1*, *msp2 *and *glurp *and required a majority of RP alleles match baseline for every locus tested.

### Genotyping markers

Once DNA was extracted, samples were genotyped using methods which relied on size and sometimes sequence variation of the genotyping markers. All markers reported were genes of polymorphic surface antigens and were amplified using the polymerase chain reaction (PCR), though different primers and conditions were often reported for the same marker. 98% of the trials for which markers were reported used the highly variable merozoite surface protein 2 (*msp2*) alone or in combination with other markers (Table 1). The *msp2 *marker has the advantage of greater genetic diversity than other commonly used markers[7], which helps to increase sensitivity for detecting new infections. Most trials used a combination of two or more markers, with all combinations including both *msp1 *and *msp2*, and with the most commonly reported combination of *msp1*, *msp2*, and glutamine-rich protein (*glurp*) used in over half of all trials. The advantage of using a combination of markers for genotyping is that it improves the ability to measure genetic diversity further, as two parasite strains may have the same allele at one marker but different alleles at another[8]. The most common method for amplifying *msp1 *and *msp2 *was nested PCR, in which the second round of PCR used primers specific for distinct families of alleles for each marker. By using family-specific primers, the ability to measure genetic diversity is increased, because alleles can be distinguished both by sequence variation between the families as well as by the size of alleles within each family. In a small proportion of trials, sequence variation in markers was assessed after PCR amplification by using family-specific hybridization probes, restriction fragment length polymorphism, direct sequencing, or a heteroduplex tracking assay.

Once markers were amplified, different alleles were identified by separating PCR products by size using electrophoresis. Though a few trials used polyacrylamide gel electrophoresis, the vast majority of trials relied on agarose, which provides lower resolution than polyacrylamide, decreasing the ability to detect genetic differences, but is faster, less expensive, and easier to perform. Many of the trials which used agarose gel electrophoresis did not specify whether comparisons between baseline and recurrent parasitaemia samples were made based on visual inspection or by comparing the measured size of alleles. Only 15% of trials specifically mentioned using a computer programme to size alleles. Of the trials which mentioned sizing alleles, the criteria used to determine whether alleles from two different samples were the same or different varied and were often not reported.

### Interpretation of results

Genotyping patterns are easy to compare when only one parasite strain, and thus one allele per marker, is present in baseline and recurrent parasitaemia samples. However, in high transmission areas such as Africa, where the majority of trials using genotyping are now performed, multiple parasite strains are often present. All trials considered an outcome recrudescence if all alleles from both samples matched and new infection if no alleles matched. However, trials varied in how they interpreted a mixed result, in which some but not all of the alleles present in the recurrent parasitaemia sample were present at baseline. 62% of trials classified mixed results as recrudescence, since at least one parasite strain may have survived therapy (Table 1). This is the more conservative definition, erring on the side of detecting recrudescence, and has been recommended for use in drug efficacy trials[8]. However, in high transmission areas when multiple alleles are present, the probability of at least one match occurring between two samples by chance may be very high, even at multiple markers, and using this conservative definition may significantly overestimate the rate of drug failure[7]. Of the trials that classified mixed results as recrudescence, 43% referenced the same paper for their methods[9]. This paper included the criterion that for an outcome to be considered recrudescence the probability of alleles from the baseline and recurrent parasitaemia samples matching by chance be less than 0.05, though it is unclear how many trials referencing this paper actually estimated this probability.

### Impact of genotyping methods on drug efficacy measures

Among the 91 antimalarial trails which used genotyping, the majority of the 175 treatment arms used either an ACT or a non-artemisinin monotherapy (Table 2). The percentage of treatment outcomes requiring genotyping would be expected to be higher for trials conducted in higher transmission areas, since new infections would be more common, and also higher with less efficacious drugs, since recrudescent infections would be more common. In Africa, where transmission intensity is high, the median percentage of subjects per arm requiring genotyping was twice as high (25%) as for trials outside of Africa (12.5%). Of the 46 trials requiring genotyping of at least 50 samples, 40 were conducted in Africa. Looking at drug class, the median percentage of subjects per arm requiring genotyping was highest for non-artemisinin monotherapies (31%) and lowest for ACTs (17%). The success of genotyping varied widely, and more than one third of arms were not successful in genotyping at least 91% of subjects for which genotyping was required (Table 2). Reasons for unsuccessful genotyping included unsuccessful amplification of parasite DNA, inability to interpret PCR results, loss of samples, and the decision to only genotype a subset of samples.

**Table 2:** Characteristics of individual treatment arms where genotyping was performed (n = 175).

**Characteristic**	**n (%)**
Drug regimen	
non-artemisinin monotherapy	68 (39%)
non-artemisinin combination therapy	29 (17%)
artemisinin monotherapy	6 (3%)
artemisinin combination therapy	72 (41%)
Total number of patients requiring genotyping *	
1–9	42 (24%)
10–19	30 (17%)
20–49	54 (31%)
≥50	30 (17%)
no samples to genotype	8 (5%)
data not available	11 (6%)
Percent of samples successfully genotyped ^†^	
≤50%	14 (9%)
51% – 90%	46 (29%)
91% – 99%	25 (16%)
100%	71 (46%)
Percent of successfully genotyped samples classified as new infections	
0%	20 (13%)
1% – 24%	25 (16%)
25% – 49%	33 (21%)
50% – 74%	41 (26%)
75% – 99%	24 (15%)
100%	12 (8%)

* Total number of patients who experienced recurrent malaria/parasitaemia excluding those patients whose recurrence occurred prior to the earliest day following therapy on which samples were collected for genotyping.

^† ^Samples not successfully genotyped include those for which genotyping was not attempted, for which genotyping failed to give a result, or for which genotyping gave indeterminate results.

The outcome measure for determining the effects of trial location and genotyping methods on results was the percent of successfully genotyped recurrences classified as new infections (Table 2). Univariate and multivariate logistic regression analyses were performed to assess the roles of five separate factors on the percent of successfully genotyped results classified as new infections: the number of markers, the interpretation of mixed results, the type of antimalarial drug studied, the geographic region in which the study took place, and the duration of follow-up. Treatment arms were classified into two outcome groups: less than 50% or at least 50% of successfully genotyped recurrences classified as new infections. Excluding eight arms with no recurrences, 11 arms without data on the number of patients who required genotyping, and 1 arm for which no recurrence was successfully genotyped, 78 arms fell into the first outcome group, and 77 fell into the second. An additional 16 treatment arms were excluded because data were not available on the number of genotyping markers used or the interpretation of mixed results, leaving 139 treatment arms included in the final analysis.

Univariate logistic regression analysis showed that both the use of combination therapy (either ACT or non-ACT) and follow-up of greater than 28 days significantly increased the odds of classifying at least 50% of successfully genotyped recurrences as new infections (Table 3). Combination therapy is generally more effective than monotherapy, and therefore would be expected to have fewer recrudescent infections, leading to a larger proportion of recurrent parasitaemia due to new infections. With longer follow up, there is more time for a new infection to occur, also increasing the proportion of recurrent parasitaemia due to new infections. When multivariate logistic regression analysis was used to account for confounding between variables, an interpretation of mixed results as not always a recrudescence and the location of the study arm in Africa were also found to significantly increase the odds of classifying at least 50% of successfully genotyped recurrences as new infections. The interpretation of mixed results as a new infection will result in more outcomes classified as new infections, though possibly missing true drug failures as discussed previously. The higher transmission in Africa compared with the rest of the world also makes new infection during follow-up more likely. The use of at least three genotyping markers was not found to increase the odds of classification as new infection in either univariate or multivariate analysis.

**Table 3:** Effect of methods on the percent of outcomes classified as new infections (n = 139).

**Explanatory Variable**	**Univariate Analysis**		**Multivariate Analysis**	
	
	OR* (95% CI)	**p-value**	OR* (95% CI)	**p-value**
Number of markers				
≥3 markers *vs*. 1–2 markers	1· 09 (0.56–2· 13)	0.80	1· 28 (0.45–3· 65)	0.64
Classification of mixed results^†^				
not always recrudescence *vs*. always recrudescence	1· 92 (0.91–4· 03)	0.09	3· 31 (1· 22–9· 01)	0.02
Treatment group				
artemisinin combination therapy *vs*. monotherapy	8· 20 (3· 52–19· 11)	< 0.0001	8· 56 (3· 34–21· 94)	< 0.0001
non-artemisinin combination therapy *vs*. monotherapy	2· 72 (1· 03–7· 16)	0.04	2· 30 (0.82–6· 47)	0.12
Geographic region				
Africa *vs*. rest of the world	0.87 (0.44–1· 71)	0.68	3· 30 (1· 01–10.84)	0.05
Maximum duration of follow-up				
> 28 days *vs*. ≤ 28 days	2· 54 (1· 23–5· 24)	0.01	4.10 (1· 28–13· 15)	0.02

* Odds Ratio for the outcome defined as: ≥ 50% of samples successfully genotyped were classified as new infections

^† ^Mixed results defined as a sample from the day of recurrent parasitaemia containing both alleles present in the baseline sample and new alleles not present in the baseline sample

## Discussion

The results of this study show that the use of genotyping for clinical antimalarial efficacy trials increased dramatically from 2001–2005, especially in Africa where the majority of these trials are now performed. There was wide variation in the methods used for all steps in the genotyping process, from the choice of subjects to genotype to the interpretation of mixed results. Trials in Africa had the highest proportion of subjects with recurrent parasitaemia, and thus the largest proportion of outcomes potentially affected by genotyping. Several factors had a significant effect on genotyping outcomes in the multivariate logistic regression analysis. The interpretation of mixed genotyping results as not always a recrudescence, the use of ACT, the location of a trial in Africa, and a follow-up duration of more than 28 days were all associated with a statistically significant increase in the number of recurrent parasitaemia episodes classified as new infections.

These data demonstrate that genotyping-corrected estimates of antimalarial efficacy from clinical trials can vary due to the setting in which genotyping is applied and the genotyping methods used. Variations in efficacy estimates may not have a major effect on treatment policy when drug failure rates are very high. However, with the introduction of more efficacious therapies such as ACT and the recent WHO recommendation that a therapy be abandoned if failure rates are greater than 10%[3], variations in efficacy estimates attributable to genotyping methods may affect policy decisions. A possible example of variation due to the setting in which genotyping was applied is seen when comparing results from four recent antimalarial trials of the ACT dihydroartemisinin plus piperaquine (DHA-PQP). This ACT has only recently become available, and there is little reason to suspect preexisting drug resistance to either component outside of China. Accordingly, three trials performed in South East Asia, where *P. falciparum *transmission is relatively low, demonstrated failure rates of 0–0.6% for this combination at 42 or 63 days [10-12]. In contrast, another study performed in Rwanda, where *P. falciparum *transmission is higher, showed higher rates of drug failure at 28 days: 1.1%, 1.3%, and 11.4% at each of three sites[13]. Rukara, the site with the highest rates of drug failure in this and a previous study[14] also had a much higher rate of new infection than the other two sites. Thus, while it is possible that the higher DHA-PQP failure rate in Rukara was due to a higher level of aminoquinoline resistance[13], another possibility is that higher transmission in Rukara led to a greater number of new infections which were misclassified as recrudescent. It is important to recognize the possibility that the accuracy of genotyping methods may vary across different settings, and this variation must be considered when comparing study results.

The WHO has made great strides in standardizing antimalarial clinical trial protocols, resulting in relatively uniform classification of treatment outcomes and aiding in the comparison of drug efficacy results between trials. The use of genotyping is now recommended for all trials[3], but both the lack of standardization of genotyping methods and the lack of criteria for appropriate methods in different geographic settings can lead to difficulty in comparing efficacy results. Variations in some aspects of genotyping, such as collection of blood on filter paper versus whole blood, are unlikely to affect outcomes. However, different interpretations of mixed results, as has been shown here and previously[5] may have a large effect on efficacy outcomes. Some studies have in the past chosen to define mixed results in recurrent parasitaemia samples as new infections in high transmission areas, as the limited resolution of genotyping methods otherwise led to too many genotyping outcomes falsely misclassified as recrudescence[7]. Ideally, however, higher resolution methods should be used so that the recurrence of even one allele can be classified as drug failure[8]. One way to improve resolution is to increase the number of genotyping markers used. In this analysis, the effect of the number of genotyping markers used was not statistically significant, possibly due to a lack of statistical power, other confounders not being assessed in the multivariate analysis, or the fact that more than three markers or markers with greater diversity are needed before better discrimination is achieved. A recent review of genotyping in two laboratories, however, showed that adding a second genotyping marker resulted in 15.5% and 18% of recrudescent infections being reclassified as new infections[15]. Greater discriminatory capacity of genotyping methods, possibly afforded by the addition of new markers such as microsatellites, may improve the ability to detect new infections in high transmission areas such as much of Africa, and should be further evaluated[16].

## Conclusion

Standard criteria for genotyping methods, including which methods provide enough discriminatory capacity for different levels of transmission and what level of genotyping misclassification is acceptable, should be decided in order to estimate drug resistance rates for *P. falciparum *more accurately and compare results between trials more easily. The need for the evaluation and standardization of genotyping methods is greatest in Africa, where the most antimalarial trials are performed, the highest percentage of outcomes need genotyping, and the interpretation of genotyping is the most difficult. The creation of one or more genotyping reference laboratories in Africa may help to establish these standards and aid in the surveillance for drug resistance and identification of the most appropriate antimalarial therapies.

## Authors' contributions

WC performed data abstraction and assisted with the statistical analysis. BG drafted the manuscript and helped with data abstraction and study design. PR assisted with study design and helped to draft the manuscript. GD conceived of the study, participated in its design and coordination, and helped to draft the manuscript. All authors read and approved the final manuscript. All authors contributed significantly to this work and declare no conflict of interest.
